# Etiological surveillance of viral diarrhea from 2017 to 2019 in Zhangzhou city, Fujian province, China

**DOI:** 10.3389/fpubh.2024.1403341

**Published:** 2024-06-11

**Authors:** Yueli Guo, Weide Chen, Guowei Wang, Huicong Yang, Qiaoling Zhou, Chunbin Zhang, Yuanjun Zeng

**Affiliations:** ^1^Collaborative Innovation Center for Translation Medical Testing and Application Technology, Department of Medical Technology, Zhangzhou Health Vocational College, Zhangzhou, Fujian, China; ^2^Department of Laboratory Medicine, Zhangzhou Affiliated Hospital of Fujian Medical University, Zhanghou, Fujian, China; ^3^Department of Obstetrics, Zhangzhou Affiliated Hospital of Fujian Medical University, Zhangzhou, Fujian, China

**Keywords:** viral diarrhea, rotavirus, norovirus, pathogen, China

## Abstract

**Background:**

Viral diarrhea is one of the major causes of morbidity and mortality in children. This study aimed to conduct etiological surveillance of viral diarrhea in Zhangzhou city, Fujian province, China, from 2017 to 2019 to identify the prevalence, distribution, and characteristics of viral pathogens causing gastrointestinal infections in the region.

**Methods:**

Stool samples were collected from patients with acute diarrhea in Zhangzhou city, Fujian province, China, from 2017 to 2019. Rotavirus, norovirus, astrovirus, and adenovirus were detected using fluorescence immunochromatography assay.

**Results:**

Of the total 5,627 samples that were collected, at least one of the viruses (rotavirus, norovirus, astrovirus and adenovirus) was found to be positive in 1,422 samples. Rotavirus, norovirus, astrovirus, and adenovirus, were detected in 53.73, 16.68, 15.52, and 14.97%, respectively. Mixed infections were determined in 17.65% of the positive samples. The predominant mixed infections observed were a combination of norovirus and astrovirus, followed by rotavirus and norovirus, and rotavirus and astrovirus. The highest positive rate was observed in the 12–23-month group for rotavirus and adenovirus, while a significantly higher positive rate was observed for norovirus and astrovirus in the 6–11-month group.

**Conclusion:**

These findings from this etiological surveillance highlight the significant burden of viral diarrhea in Zhangzhou city, with rotavirus being the predominant pathogen. The identification of common mixed infections provides insights into the complex nature of viral diarrhea transmission. Target interventions and public health strategies should be implemented, particularly during the winter and spring seasons, to prevent and control the spread of viral pathogens causing gastrointestinal infections in this region.

## 1 Introduction

Diarrhea disease is one of the leading causes of morbidity and mortality among children in developing countries (1, 2). Globally, diarrhea causes 446,000 deaths among children under the age of five annually, accounting for nine percent of overall child mortality (3). Although sanitation improvements and vaccine introduction have significantly reduced the incidence of diarrhea, it remains one of the top five causes of death among under-five children in the world (4). Therefore, monitoring diarrheal diseases is still a priority (5).

Acute diarrhea could be attributed to a variety of factors, such as viral, bacterial and parasitic pathogens, with viral infection being the most common one (5). Approximately 75% of diarrheal cases were caused by viral pathogens (6, 7), with common etiological agents including rotavirus (RV), norovirus (NV), astrovirus (AstV), and adenovirus (AdV) (8). Among these viruses, rotavirus and norovirus are the primary culprits for acute diarrhea in children, while adenovirus and astrovirus are also known to cause sporadic diarrhea and outbreaks in pediatric populations (9).

Of all the viruses that cause diarrhea, licensed vaccines are available for only rotavirus (10). Given the high burden of rotavirus gastroenteritis worldwide, the World Health Organization recommended that rotavirus vaccines be included in all national vaccination programs. There are two licensed rotavirus vaccines widely used: RotaTeq (a pentavalent rotavirus vaccine) and Rotarix (a monovalent rotavirus vaccine). These two vaccines have proven effective in reducing the incidence of RV-related hospitalization and mortality (11). To date, over 100 countries included rotavirus vaccines in their national immunization program (12). In China, rotavirus vaccines were not included in the Chinese national immunization programs (NIP) and vaccination was voluntary. Previously, only a locally manufactured, lamb rotavirus G10P (12) strain vaccine, Lanzhou Lamb rotavirus (LLR) vaccine (Lanzhou Institute of Biological Products, Lanzhou, China) was licensed since 2000, but the LLR vaccination coverage is low (13). Hence, in order to control rotavirus infection, RotaTeq vaccines have been available on the market since 2018. As the coverage of rotavirus vaccine expands, it may lead to decrease in diarrhea caused by viral pathogens like rotavirus (14). Thus, other pathogens of viral diarrhea such as norovirus, astrovirus, and adenovirus require more attention (15).

Currently, China is still one of the 15 high diarrhea burden countries (16). Previous studies have demonstrated that the prevalence and characterization of viral diarrhea varies by location and changes over time (16, 17). In general, infections were predominantly detected in winter and spring. Rotavirus and norovirus are the two leading viral pathogens detected in the patients, followed by adenovirus and astrovirus (16). The most vulnerable population are children under 5 years of age. Although there are currently many studies that investigated the gastrointestinal pathogens in acute diarrhea (18–22) the related research in Zhangzhou city, Fujian province is still limited. Situated in the southeastern coastal area of China, Zhangzhou city experiences a distinct climate and environmental conditions that may influence the transmission and distribution of viral pathogens causing diarrheal diseases. A better understanding of the etiology, epidemiology and seasonality of acute diarrhea was of great importance for planning and adopting targeted preventive measure, as well as clinical therapy. Here, we conducted a surveillance study over 3 years in patients with acute diarrhea in Zhangzhou to inform polices and interventions targeted at preventing childhood diarrhea (23).

## 2 Materials and methods

### 2.1 Study population

From July 2017 to November 2019, continuous monitoring of acute diarrhea cases among patients was carried out at the Zhangzhou Affiliated Hospital of Fujian Medical University. Acute diarrhea was defined as ≥3 loose, watery, mucus-, or bloody-stools within 24 h (16). The study focused on children under the age of 14 who presented with diarrhea, encompassing both outpatient and inpatient data. Outpatient cases referred to those who receive medical treatment at a hospital without requiring overnight admission, while inpatients cases were defined as patients who required admission and stayed in the hospital at least an overnight. All subjects were divided into six age groups: 0–11, 12–24, 25–36, 37–48, 49–60, and older than 60 months.

### 2.2 Specimen collection

Stool samples were collected and aliquoted into feces collection tubes. The samples were sent to the laboratory of Zhangzhou Affiliated Hospital of Fujian Medical University within 24 h and stored in a freezer at −80°C until use.

### 2.3 Laboratory detection of stool samples

Four viruses including rotavirus, norovirus, astrovirus and adenovirus were identified using a rapid fluorescence immunochromatographic assay provided by Beijing Bohui Innovation Technology Co., Ltd. The detection process was strictly conducted according to the manufacturer's instructions. Briefly, 100 μL of liquid stools or 50–100 mg of solid stools were added to 1 mL of sample dilution buffer, vortexed until homogenized. And then four drops of the diluted stool specimen were added to the rapid detection cards. Subsequently, the cards were placed in the Fluorescence immune-quantitative analyzer (BH400, Bohui Innovation Technology Co., Ltd) for analysis 5–8 min later. Fluorescence values were measured according to the instruction.

### 2.4 Statistical analysis

The required sample size was calculated by using the *MedSci* Sample Size tools (MSST). According to the literature, the incidence of viral diarrhea was about 25% and the allowable error (δ) was 2%. If we considered α = 0.05, the required sample size was 1,801. All statistical analysis was performed using GraphPad Prism 10.1.2 software (GraphPad Software). Chi square test was used for categorical variables. A *p*-value < 0.05 was considered statistically significant.

## 3 Results

### 3.1 Basic information

**Figure 1:**
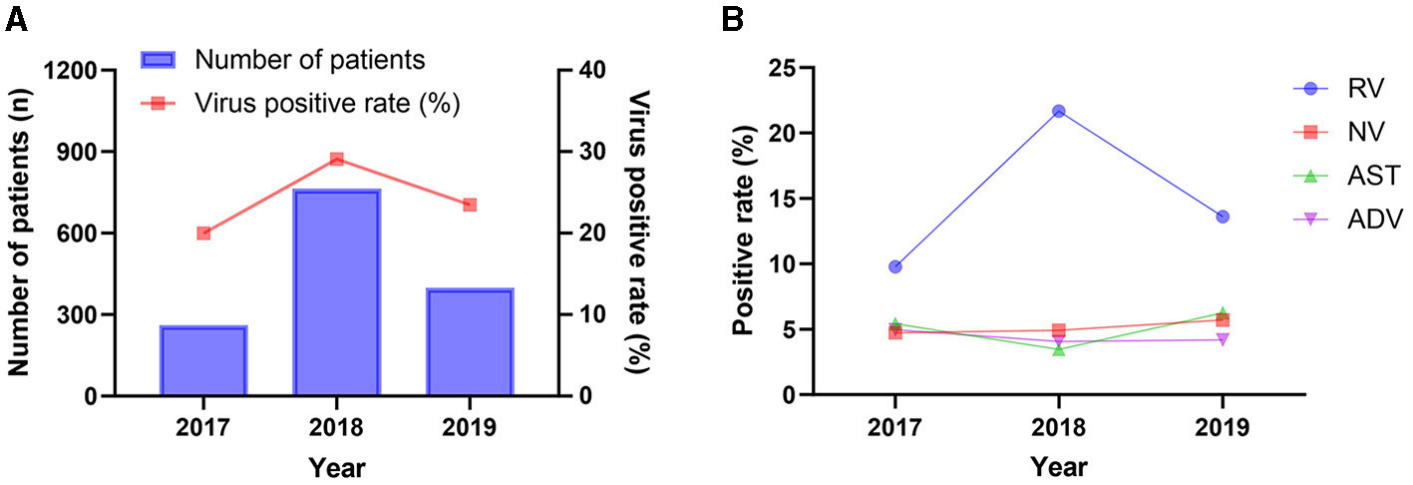
Positive rates of the diarrheal infections in different year. **(A)** The overall detection of diarrheal infections; **(B)** Distribution of different diarrhea viruses during 2017–2019.

From July 2017 to November 2019, a total of 5,627 samples collected from pediatric patients presenting with acute diarrhea were included in a study conducted in Zhangzhou city, Fujian province, China. The samples were distributed as follow: 1,307 samples in 2017, 2,624 samples in 2018, and 1,696 samples in 2019. The demographic and epidemiological characteristics of the studied patients are shown in Table 1. The overall positive rate remained relatively stable, with a slight increase observed from 2017 (19.97%, 261/1,307) to 2018 (29.08%, 762/2,624), followed by a slight decrease in 2019 (23.47%, 398/1,696). A similar trend was noted for the positive rate of rotavirus, as shown in Figure 1. Of whom 62.54% (3,519/5,627) were male, and 91.06% (5,124/5,627) were under the age of 5 years. A higher proportion of patients were recruited from the inpatient department (64.49%, 3,629/5,627) compared to the outpatient department (Table 1).

**Table 1:** Characteristics of patients with acute diarrhea in Zhangzhou, 2017–2019.

**Characteristics**	**All virus tested (*n* = 5,627)**	**Any virus positive (*n* = 1,422)**	**Co-infection (*n* = 251)**
**Sex**			
Male	3,519 (62.54)	869 (61.11)	161 (64.14)
Female	2,108 (37.46)	553 (38.89)	90 (35.86)
**Age group (months)**			
0–11	2,056 (36.54)	358 (25.18)	77 (30.68)
12–23	1,739 (30.90)	494 (34.74)	86 (34.26)
24–35	745 (13.24)	317 (22.29)	45 (17.93)
36–47	385 (6.84)	103 (7.24)	16 (6.37)
48–59	199 (3.54)	56 (3.94)	9 (3.59)
≥60	503 (8.94)	94 (6.61)	18 (7.17)
**Case type**			
Outpatients	1,998 (35.51)	477 (33.54)	94 (37.45)
Inpatients	3,629 (64.49)	945 (66.46)	157 (62.55)

Data are n (%) unless otherwise indicated. Constituent ratio of pathogens that caused acute diarrhea (%).

### 3.2 Gender distribution

Among the 5,627 cases, 3,519 were males and 2,108 were females, and the male to female was 1.67:1. The detection rates by gender were 24.69% (869/3,519) in males and 26.23% (553/2,108) in female (Table 1). The difference between them was not statistically significant (*p* = 0.199). Among the co-infected patients, 161 were males and 90 were females (Table 1). The co-infection rates were 18.53% (161/869) and 16.27% (90/553) in males and females, respectively, with no significant difference between genders (*p* = 0.278).

### 3.3 The age-specific pattern of viral infection

**Figure 2:**
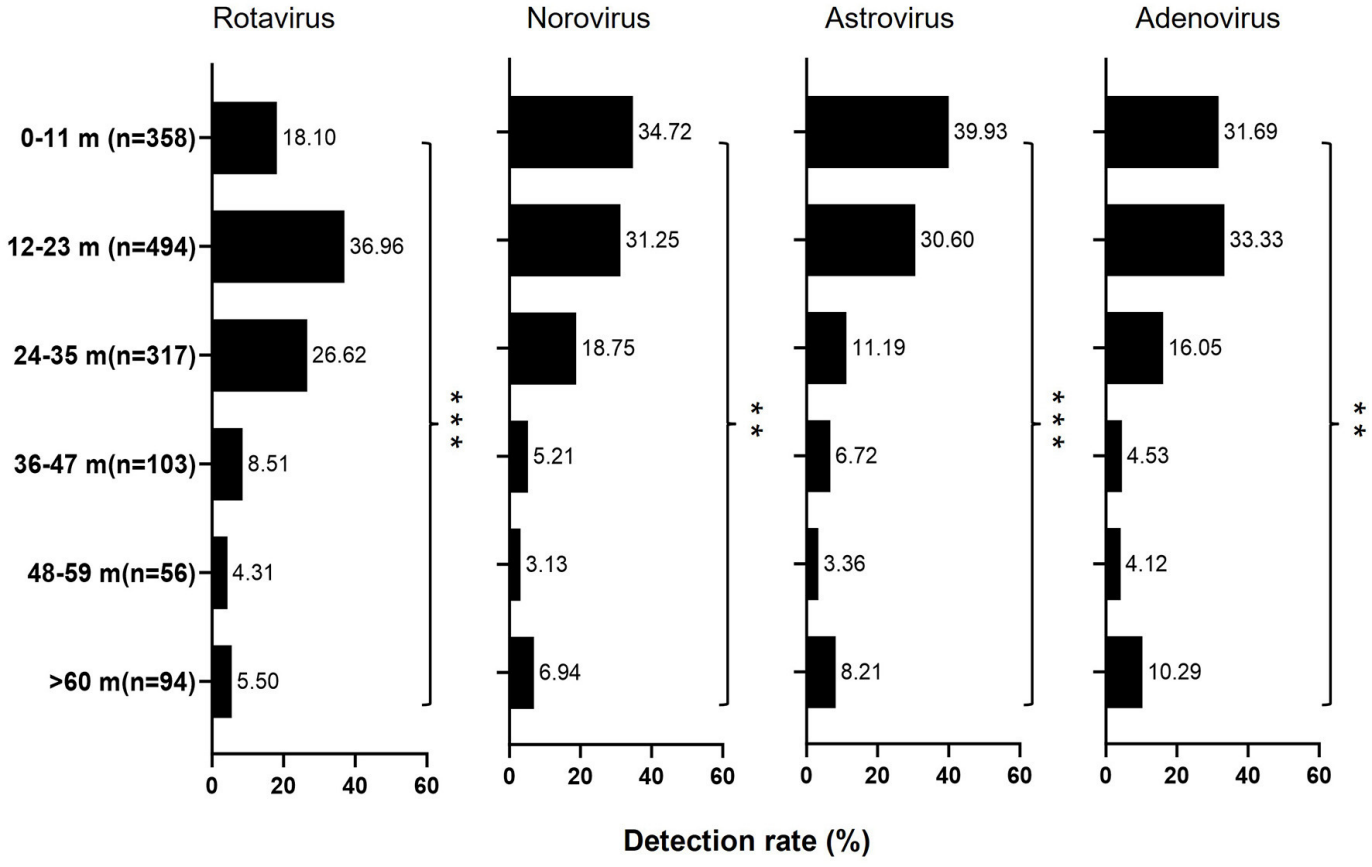
Distribution of the positive subjects (*n* = 1,422) by age class and diarrhea virus identified. The length of color bars indicated the detection rate of each pathogen by age. The Chi-square test was used for comparison among age groups (*p < 0.05; **p < 0.01; ***p < 0.001).

All the enteric viruses were determined in all age groups, however, their infection patterns varied by age. As summarized in Table 1, when all patients were analyzed together, the positive rate increased rapidly with age, peaking at age 12–23 months and then decreased (Chi-square test, *p* < 0.001). Moreover, a significantly higher viral positive rate was observed in 12–23 months old children (34.74%, 494/1,422) than in other age groups, especially with a higher rate of rotavirus (36.96%, 343/928) and adenovirus (33.33%, 81/243). In contrast, children aged 0–11 months displayed a significantly higher positive rate for norovirus (34.72%, 100/288) and astrovirus (39.92%, 107/268; Figure 2 and Supplementary Table 1).

### 3.4 Pathogenic spectrum of viral diarrhea

**Figure 3:**
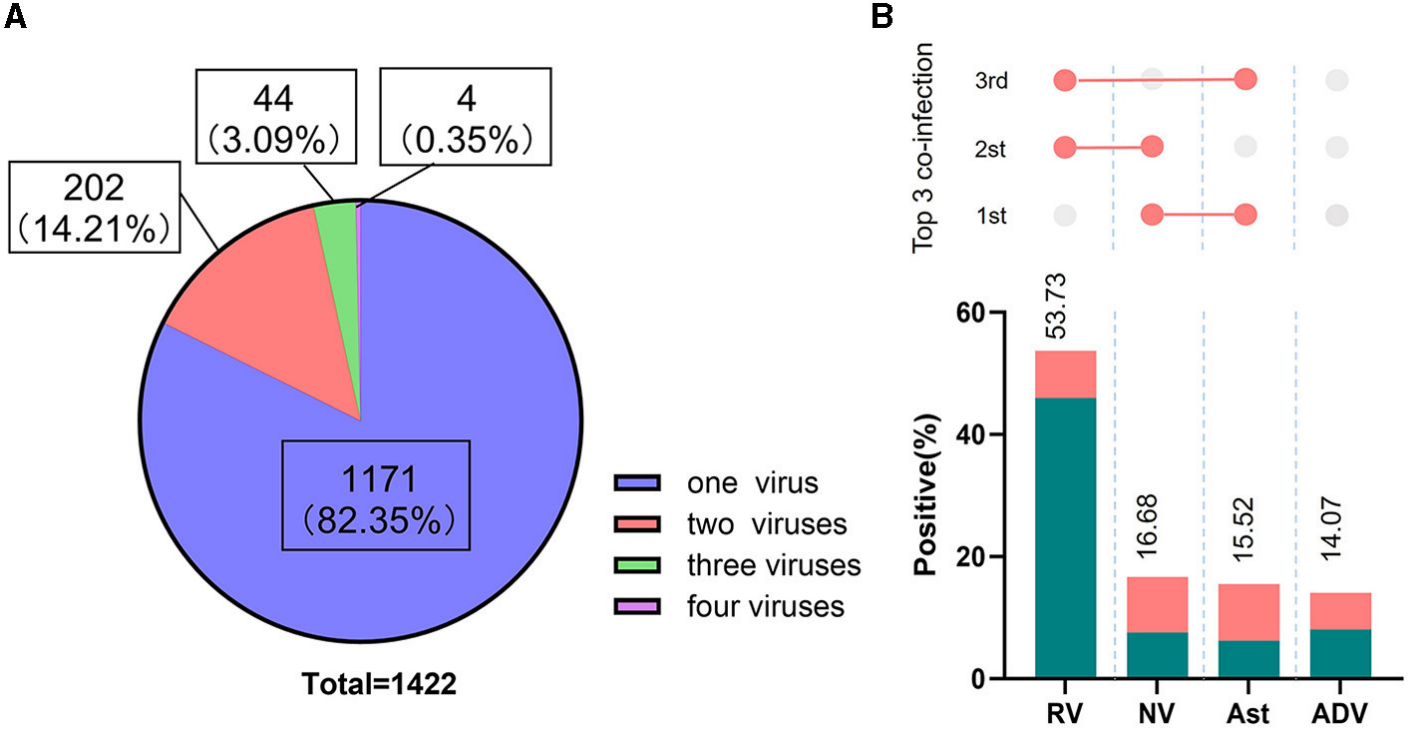
Proportion of co-infections and viruses identified. **(A)** Graphical description of single and multi-virus infections in positive stools; **(B)** The percentage of the most common co-infection patterns detected. The proportion of each positive pathogen was noted in % and by the length of colored bars. The green bar indicates mono-infection, the orange bar indicates co-infections. The three most common co-infections were presented above the constituent ratios.

Among the 5,627 samples tested for four kinds of viruses, 1,422 tested positive (25.27%, 1,422/5,627). As shown in Figure 3A, 1,171 samples were infected with on pathogen, 202 samples were infected with two pathogens, 44 samples were infected with three pathogens, and 5 samples were infected with four pathogens, with mixed infections accounting for 17.65% of positive specimens (251/1,422). The percentages of positive samples for rotavirus, norovirus, astrovirus, and adenovirus to the total positive samples were 53.73, 16.68, 15.52, and 14.07%, respectively (Figure 3B). The top-ranking co-infection occurred between norovirus and astrovirus, the second most common co-infection occurred between norovirus and rotavirus, and the third co-infection occurred between rotavirus and astrovirus (Figure 3B).

### 3.5 Temporal/seasonal distribution of the four pathogens causing infectious diarrhea

**Figure 4:**
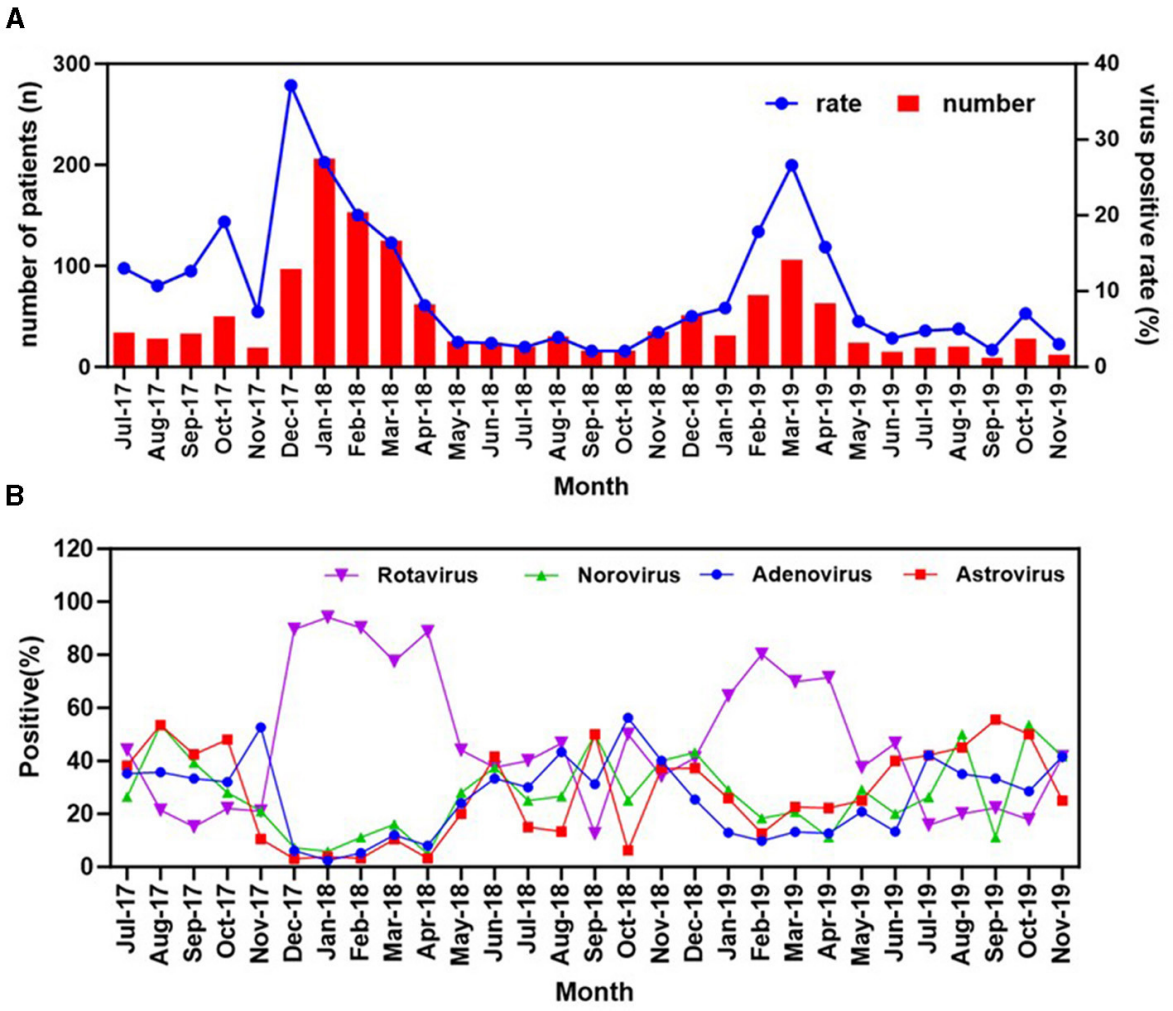
Seasonal distribution of the diarrheal infections and the four enteric viruses detected in stools. **(A)** The annual trend of the cases of acute diarrhea and the cumulative positivity rate for the four viruses. **(B)** Monthly distribution of the four viruses among diarrhea patients in Zhangzhou, Fujian Province, China from 2017 to 2019.

During the study period from 2017 to 2019, the overall positive rate varied with month and season of sample collection. As seen in the Figure 4A, a clear seasonal pattern was observed for the overall positive rate, with a higher level of circulation observed in the winter (38.04%, 609/1,601) and spring (31.57%, 405/1,283) compared to the summer (17.23%,190/1,103) and autumn (13.29%, 218/1,640; *p* < 0.001, Table 2). This trend was particularly pronounced in rotavirus (Figure 4B), which emerged as the predominant cause of viral diarrhea during these colder seasons. No significant trends were observed for norovirus when each of the tested viruses was separately analyzed. The low detection rate for the virus may account for this observation.

**Table 2:** The virus detection rates for different seasons in pediatric patients with acute diarrhea.

**Viruses**	**Spring (*n* = 1,283)**	**Summer (*n* = 1,103)**	**Autumn (*n* = 1,640)**	**Winter (*n* = 1,601)**	***P*-value**
Any virus	405 (31.57)	190 (17.23)	218 (13.29)	609 (38.04)	<0.001
Rotavirus	291 (22.68)	66 (5.98)	54 (3.29)	517 (32.29)	<0.001
Norovirus	66 (5.14)	64 (5.80)	78 (4.76)	80 (5.00)	0.669
Astrovirus	64 (4.99)	68 (6.17)	84 (5.12)	52 (3.25)	0.004
Adenovirus	53 (4.13)	66 (5.98)	81 (4.94)	43 (2.69)	<0.001

Data are n (%) unless otherwise indicated.

It is noteworthy that a clear relationship was found between the increase in rotavirus cases and a decrease in the prevalence of other enteric viruses. Specifically, during the peak months of winter and early spring, rotavirus was responsible for more than 60% of all viral diarrhea cases, while the combined incidence of other enteric viruses, such as adenovirus, norovirus, and astrovirus, was substantially lower. This pattern remained consistent across multiple years of data collection, indicating a seasonal trend rather than a random fluctuation.

## 4 Discussion

Infectious diarrhea is an important global public health problem, and viral diarrhea is an important part of infectious diarrhea (24). It is imperative to conduct thorough and ongoing surveillance of viral pathogens to inform effective public health interventions. Hence, in order to gain a more comprehensive understanding of the spectrum of viral diarrhea and its changing trends in Zhangzhou city more comprehensively, four kinds of virus infection in patients with infectious diarrhea were monitored from 2017 to 2019.

Among virus-positive specimens, rotavirus exhibited the highest positive detection rate, followed by norovirus, astrovirus, and adenovirus (Figure 3). The predominance of rotavirus aligns with global trends, as it is widely acknowledged as one of the leading causes of severe gastroenteritis in children worldwide (25). This highlights the prevalence and burden of rotavirus and underscores the importance of continued efforts to prevent, diagnose and treatment of rotavirus diarrheal disease. Additionally, our results reveal a fluctuation in the detection of rotavirus across the study years (Figure 1), the possible explanations for this may be as follows: firstly, samples were not obtained during January and June 2017, which could lead to underestimates of test percentage positivity; secondly, the decrease in the positive rate for rotavirus in 2019 compared to 2018 may be related to the introduction of the rotavirus vaccine RotaTeq in 2018. Further research is required to assess the long-term protective efficacy of this vaccine.

To be noted, the result revealed a significant incidence of co-infections (17.65%, 251/1,422), particularly between norovirus and astrovirus, followed by rotavirus and norovirus, as well as rotavirus and astrovirus. This finding contrasts with previous studies, which identified co-infection with rotavirus and norovirus as the most common (16, 26). The underlying reasons for this discrepancy warrant further investigation. The significant occurrence of co-infections underscores the complexity of viral diarrhea etiology and the necessity for diagnostics tools capable of identifying multiple pathogens to inform appropriate treatment and control measures.

The age distribution of detected viral pathogens, predominantly in children aged 12–23 months, underscores the vulnerable nature of this age group to viral diarrheal infection. This age-specific pattern is consistent with previous research underlining the importance of targeted interventions, such as rotavirus vaccination initiatives, to alleviate the impact of these illnesses within this demographic.

The observed seasonal trend in the incidence of rotavirus-associated diarrhea aligns with finding from previous studies, suggesting the prominent role of rotavirus in viral diarrhea during colder months (23, 26). The decrease in the prevalence of other enteric viruses during the peak of the rotavirus season suggests a potential competitive exclusion mechanism, wherein the high prevalence of rotavirus may inhibit the transmission of other viruses. Future researches should aim to elucidate the mechanisms driving the seasonal patterns and assess the effectiveness of various prevention and control strategies.

There are some limitations to this study. Firstly, the study focused only on the four viruses, assuming that all samples were negative for parasites and bacteria. However, this approach may have overlooked other potential viral or non-viral causes of diarrhea, thereby limiting our understanding of the complete etiology of diarrhea in the region. Secondly, the study failed to gather data on the rotavirus vaccination status of participants, which could have significantly influenced the incidence and severity of rotavirus infection. Lastly, the absence of clinical data, including details on the severity and symptoms of the diarrhea cases, further limits the depth of analysis in this study. These constraints suggests that future studies should incorporate a wider range of pathogens, detailed vaccination histories, and comprehensive clinical data to provide a more complete understanding of the factors influencing viral diarrhea.

## 5 Conclusion

In conclusion, the primary viral agent responsible for diarrhea in Zhangzhou was identified as rotavirus, with the other three viruses (norovirus, astrovirus, and adenovirus) also being detected. Notably, the incidence rates were higher during the chiller winter and spring. In the future, surveillance will be further strengthened to carry out prototype and epidemiological investigation of viral diarrhea disease, in order to play a practical role in the prevention and control of regional viral diarrhea and early warning.

## Data availability statement

The original contributions presented in the study are included in the article/Supplementary material, further inquiries can be directed to the corresponding author.

## Ethics statement

The studies involving humans were approved by the Ethics Committee of Zhangzhou Affiliated Hospital of Fujian Medical University. The studies were conducted in accordance with the local legislation and institutional requirements. The Ethics Committee/Institutional Review Board waived the requirement of written informed consent for participation from the participants or the participants' legal guardians/next of kin because of its retrospective study design.

## Author contributions

YG: Conceptualization, Writing – original draft. WC: Investigation, Methodology, Writing – original draft. GW: Investigation, Methodology, Writing – original draft. HY: Formal analysis, Writing – original draft. QZ: Formal analysis, Writing – original draft. CZ: Resources, Writing – original draft. YZ: Conceptualization, Methodology, Writing – review & editing, Writing – original draft.
