# Supplementary material for: Etiological surveillance of viral diarrhea from 2017 to 2019 in Zhangzhou city, Fujian province, China
Source: Front Public Health. 2024 Jun 11;12:1403341. doi: 10.3389/fpubh.2024.1403341 (PMC11196759; doi:10.3389/fpubh.2024.1403341)
Supplement: Supplementary file 1 [file Table_1.DOCX]

**Tabel S1.** The virus detection rates for different seasons in pediatric patients with acute diarrhea

| viruses | Age group (month) | | | | | | *P* value |
| --- | --- | --- | --- | --- | --- | --- | --- |
|  | 0-11 | 12-23 | 24-35 | 36-47 | 48-59 | >60 |  |
| Rotavirus (n=928) | 168 (18.10) | 343 (36.96) | 247 (26.62) | 79 (8.51) | 40 (4.31) | 51 (5.50) | < 0.001 |
| Norovirus (n=288) | 100 (34.72) | 90 (31.25) | 54 (18.75) | 15 (5.21) | 9 (3.13) | 20 (6.94) | 0.002 |
| Astrovirus (n=268) | 107 (39.93) | 82 (30.60) | 30 (11.19) | 18 (6.72) | 9 (3.36) | 22 (8.21) | <0.001 |
| Adenovirus (n=243) | 77 (31.69) | 81 (33.33) | 39 (16.05) | 11 (4.53) | 10 (4.12) | 25 (10.29) | 0.002 |
